# Computational design of *Lactobacillus Acidophilus* α-L-rhamnosidase to increase its structural stability

**DOI:** 10.1371/journal.pone.0268953

**Published:** 2022-05-25

**Authors:** Thassanai Sitthiyotha, Methus Klaewkla, Kuakarun Krusong, Rath Pichyangkura, Surasak Chunsrivirot

**Affiliations:** 1 Structural and Computational Biology Research Unit, Department of Biochemistry, Faculty of Science, Chulalongkorn University, Pathumwan, Bangkok, Thailand; 2 Department of Biochemistry, Faculty of Science, Chulalongkorn University, Pathumwan, Bangkok, Thailand; Bioinformatics Institute, SINGAPORE

## Abstract

α-L-rhamnosidase catalyzes hydrolysis of the terminal α-L-rhamnose from various natural rhamnoglycosides, including naringin and hesperidin, and has various applications such as debittering of citrus juices in the food industry and flavonoid derhamnosylation in the pharmaceutical industry. However, its activity is lost at high temperatures, limiting its usage. To improve *Lactobacillus acidophilus* α-L-rhamnosidase stability, we employed molecular dynamics (MD) to identify a highly flexible region, as evaluated by its root mean square fluctuation (RMSF) value, and computational protein design (Rosetta) to increase rigidity and favorable interactions of residues in highly flexible regions. MD results show that five regions have the highest flexibilities and were selected for design by Rosetta. Twenty-one designed mutants with the best ΔΔG at each position and ΔΔG < 0 REU were simulated at high temperature. Eight designed mutants with ΔRMSF of highly flexible regions lower than -10.0% were further simulated at the optimum temperature of the wild type. N88Q, N202V, G207D, Q209M, N211T and Y213K mutants were predicted to be more stable and could maintain their native structures better than the wild type due to increased hydrogen bond interactions of designed residues and their neighboring residues. These designed mutants are promising enzymes with high potential for stability improvement.

## Introduction

α-L-Rhamnosidase (E.C. 3.2.1.40) is a member of glycoside hydrolase family 78 (GH78). The structure of α-L-rhamnosidase consists of one α-domain (domain A) and four β-domains (domains N, E, F and C). Domain A, which has an (α/α)6-barrel structure, is the catalytic module, while domains N, E, F and C consist almost solely of β-strands and surround the catalytic module. α-L-Rhamnosidase catalyzes hydrolysis of the terminal non-reducing α-L-rhamnosyl residues from a large number of natural rhamnoglycosides including naringin, rutin, diosgene, hesperidin, terpenyl glycosides [1]. α-L-Rhamnosidase can be found in many microorganisms such as *Aspergillus niger* [2], *Aspergillus terreus* [3], *Bacillus sp*. [4], *Clostridium stercorarium* [5], *Lactobacillus acidophilus* [6], *Lactobacillus plantarum* [6], *Penicillium aurantiogriseum* [7], *Streptomyces avermitilis* [8] and *Trichoderma longibrachiatum* [7]. Due to its various potential applications, α-L-rhamnosidase is an important enzyme in many biotechnological processes. For example, it can be used to hydrolyze the terpenyl glycosides containing L-rhamnose to enhance the wine aromas [9, 10]. Derhamnosylation by this enzyme can enhance the pharmacological properties of some drugs, such as steroids, ruscin and gypenosides [11–13]. α-L-Rhamnosidase also eliminates the hesperidin crystals from orange juices [14]. Additionally, it can catalyze hydrolysis of naringin in debittering and clearance of citrus fruit juices [15, 16].

**Fig 1:**
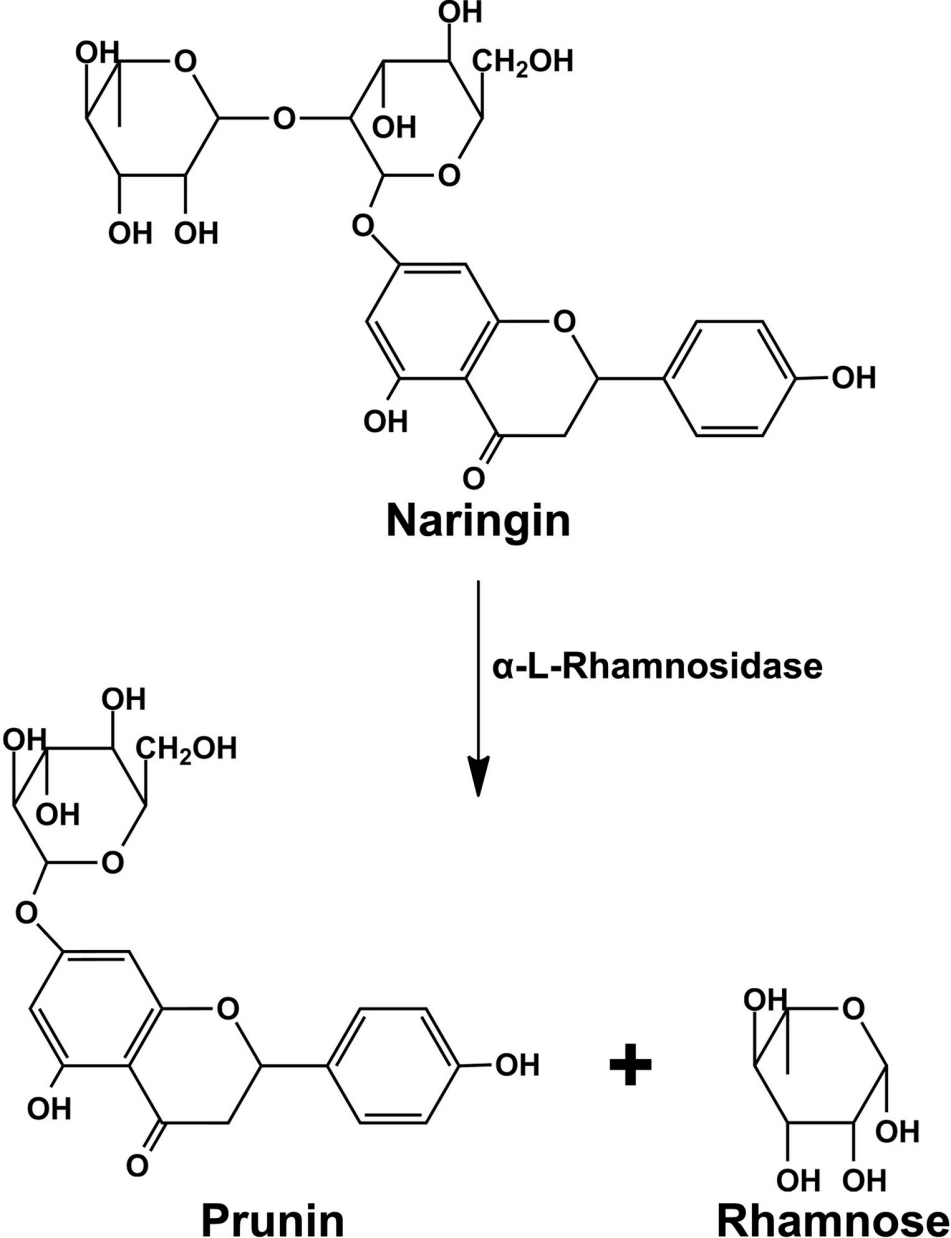
α-L-rhamnosidase catalyzes the hydrolysis of naringin to prunin and rhamnose.

Naringin (NAR) is a flavanone glycoside consisting of the flavanone naringenin and the disaccharide neohesperidose. It is one of the main active components of several medicinal plants and fruits. Naringin is also found in citrus fruits and provides a bitter taste to citrus fruits juices. Naringin is hydrolyzed by α-L-rhamnosidase to produce prunin (Fig 1), which is subsequently converted to naringenin by the hydrolysis of β-D-glucosidase. Therefore, α-L-rhamnosidase can potentially be used to reduce the bitter taste of citrus fruits juices. Similar to other flavonoids, Naringin and naringenin have various pharmacological activities including anti-cancer activities, anti-inflammatory, antioxidant and anti-carcinogenic properties. Additionally, these compounds also have beneficial effects on many central nervous system (CNS) diseases, including Alzheimer’s disease and Parkinson’s disease [17, 18].

Enzymes have emerged as important biocatalysts in many industrial processes, especially in food and pharmaceutical industries [19, 20]. Enzymes are also preferred to chemical catalysts at high temperature. However, many enzymes lost their activities at high temperature, limiting their durations of usage [21]. Fruit juice production also requires high temperature in some steps [22], and *Lactobacillus acidophilus* α-L-rhamnosidase cannot catalyze the hydrolysis of naringin at its optimum temperature of about 40°C for a long period of time due to the loss of its activity and stability. Therefore, increasing the thermostability of *Lactobacillus acidophilus* α-L-rhamnosidase is very important for its usage. Protein flexibility is an important factor affecting enzyme thermostability. Highly flexible residues tend to cause protein unfolding and denaturation, leading to reduction of enzymatic activity [23]. One of effective approaches to improve enzyme thermostability is rigidifying a highly flexible region by increasing rigidity and various interactions such as hydrogen bonds, salt-bridges and hydrophobic interactions of amino acid residues in a highly flexible region. This approach is based on the knowledge that thermophilic proteins can preserve their native folding and structural rigidity at high temperature because of various favorable interactions between amino acid residues [24–26].

Molecular dynamics (MD) simulations can be employed to identify highly flexible regions and accelerate protein unfolding at high temperature. The flexibility of each residue is evaluated by its Root Mean Square Fluctuation (RMSF) value. Computational protein design is a promising method that can be used to improve enzyme thermostability by substituting residues in a highly flexible region with amino acids that can form favorable interactions with their neighboring residues. Moreover, the substitution of amino acid residues on the surface of an enzyme is more preferable to those in its core structure because these mutations have less detrimental effects on enzymatic activity [27, 28]. This approach has been employed to successfully improve the thermostabilities of many enzymes such as *Bacillus circulans* xylanase [28], *Bacillus licheniformis* RN-01 levansucrase [29, 30], *Escherichia coli* AppA phytase [27], *Pseudomonas putida* NRRL-18668 nitrile hydratase [31], bacterial α-Carbonic anhydrase [32] and aldo-keto reductases [33]. Furthermore, computational techniques were also employed to improve the thermostability of fungal α‐L‐rhamnosidase [34].

The aim of this work is to use MD (AMBER) and computational protein design (Rosetta) to improve the structural stability of α-L-Rhamnosidase from *Lactobacillus acidophilus*. The highly flexible regions on the enzyme surface, which can be important for thermostability and maintaining enzymatic activity, are identified based on their RMSF values by MD at high temperature to accelerate unfolding, and they are used as designed regions. The residues in the designed regions were allowed to be any of standard amino acids to increase their rigidity and favorable interactions with their neighboring residues. The best designed mutants were subjected to MD validation at high temperature and optimum temperature of the wild type to validate whether their designed regions have lower flexibility than the wild type, based on their RMSF values. The designed mutants with decreased flexibilities of the highly flexible regions are promising enzymes with high potential for stability improvement, and they were further analyzed in terms of secondary structure and hydrogen bond interactions.

## Methods

### Structure preparation

SWISS-MODEL server [35–38] was employed to build three homology models of α-L-rhamnosidase from *Lactobacillus acidophilus*. Model A (identity = 30.15% and coverage = 90.74%) and B (identity = 34.38% and coverage = 86.54%) were constructed using the crystal structure of *Dictyoglumus thermophilum* α-L-rhamnosidase (PDB ID: 6I60 [39]) as the template, and they have the highest coverage and identity, respectively. Model C (identity = 30.38% and coverage = 90.53%) was built using the crystal structure of *Streptomyces avermitilis* α-L-rhamnosidase (PDB ID: 3W5M [40]) as the template. Ramachandran plots were used to evaluate the qualities of these models. As shown in S1 Fig in S2 File, the majority of amino acid residues of all models are in favored region (Model A: 90.0%, Model B: 88.2% and Model C: 89.8%) and allowed region (Model A: 7.7%, Model B: 9.2% and Model C: 7.9%), indicating the reasonable qualities of these constructed homology model. Moreover, the positions of the catalytic residues (Glu442 and Glu712) of these homology models are similar to those of the templates. All ionized amino acids were protonated at the experimental pH 6.0 using H^++^ server [41].

### MD simulations

Using protein ff14SB force field parameters [42] in AMBER18 [43], each model was immersed in an isometric truncated octahedral box of TIP3P water molecules with the buffer distance of 13 Å. The five-step minimization procedure [29, 30, 44–56] was performed to reduce unfavorable interactions of each model. All steps used 2,500 steps of steepest descent and 2,500 steps of conjugate gradient with different restraints on the proteins. The heavy atoms of protein were initially restrained with a force constant of 10 kcal/ (mol Å^2^), while water molecules and hydrogen atoms were minimized. Subsequently, the backbone atoms of the proteins were restrained with the force constants of 10, 5 and 1 kcal/ (mol Å^2^), respectively. In the last step, the entire system was minimized without any restraining force. Then, all systems were simulated under the periodic boundary condition using the GPU (CUDA) version of PMEMD module [57–59]. The SHAKE algorithm [60] was used to constrain all bonds involving hydrogen atoms, allowing the time step of 0.002 ps. The Langevin dynamics technique [61] was employed to control the temperatures of all simulations with a collision frequency of 1.0 ps^-1^. The system was heated in the NVT ensemble from 0 K to 313 K (the optimum temperature of this enzyme) for 200 ps, while the backbone atoms of proteins were restrained with a force constant of 10 kcal/ (mol Å^2^). The system was then equilibrated in the NVT ensemble with no restraining force for 300 ps. Finally, the system was simulated in the NPT ensemble at 313 K and 1 atm for 80 ns. To analyze the structural stability during the simulations of each system, Root Mean Square Deviation (RMSD) values were calculated as shown in S2 Fig in S2 File. The trajectories of all models were found to be stable in the range of 60 to 80 ns based on their RMSD values. The centroid structure of each model, which is the structure closest to the average structure of the 60–80 ns trajectory, was selected to be a presentative structure of each model. To increase structural movements to effectively identify highly flexible regions with high RMSF values, the centroid structure of each model was minimized and simulated at 500 K for 10 ns, using similar minimization and MD simulation procedures.

### Identification of highly flexible residues

All trajectories of simulations at 500 K of each model were used to calculate the Root Mean Square Fluctuation (RMSF) values to identify the flexibility of each residue. To determine a cutoff for determining a highly flexible residue, the average RMSF values of all residues of all models were calculated, and highly flexible residues were defined as residues with RMSF values higher than the cutoff. The highly flexible residues with their adjacent highly flexible residues were then combined to create highly flexible regions. Five highly flexible regions (region 2, 3, 4, 8 and 11) with the highest average RMSF value were selected for computational protein design.

### Computational protein design and MD simulations of designed mutants

The centroid structure of the molecular dynamics trajectory at 313 K of model A was used as a design template. Each highly flexible residue was designed by allowing it to be any of standard amino acids that could potentially form favorable interactions with neighboring residues. The CoupledMoves protocol [62] in RosettaDesign module of Rosetta3.11 [63] with beta_nov16 energy function was employed to design, repack, and minimize the structure of each designed residue. The neighboring residues within 10 Å of the design position were also repacked and minimized. For each design, 400 independent runs were performed, resulting in the total of 400 conformations of designed sequences (some sequences might have multiple conformations). The free energy of each conformation of each designed sequence (ΔG_mutant_) was calculated in Rosetta Energy Unit (REU). The value of ΔΔG of each designed sequence/conformation was calculated from the difference between the free energy of the designed sequence/conformation (ΔG_mutant_) and the free energy of the wild type (ΔG_wild type_) (ΔΔG = ΔG_mutant_—ΔG_wild type_). The designed sequences/conformations with ΔΔG values of its designed position < 0 REU were selected for MD simulations at 500 K for 10 ns in triplicate. The RMSF values of the highly flexible regions of the selected designed mutants were analyzed to determine whether their highly flexible regions have lower flexibility than the wild type. The designed mutants with ΔRMSF values of the highly flexible regions lower than -10.0% were further simulated at 313 K (the optimum temperature of the wild type) for 80 ns to validate whether their highly flexible regions have lower flexibility than the wild type, based on their RMSF values.

### Hydrogen bond interaction analysis

Hydrogen bond interactions of the designed mutants and wild type were analyzed to determine the interactions that are crucial for improved stability of the designed mutants. Hydrogen bond interactions between all residues in the highly flexible regions and their neighboring residues were determined. Hydrogen bond occupations were then calculated to analyze hydrogen bond interactions, a hydrogen bond was considered to occur if the following criteria were met: (i) a proton donor-acceptor distance ≤ 3.5 Å and (ii) a donorH-acceptor bond angle ≥ 120° [44, 45, 53, 55]. The strengths of hydrogen bond interactions were classified into four levels depending on hydrogen bond occupations: (i) strong hydrogen bond interactions (hydrogen bond occupations > 75%) (ii) medium hydrogen bond interactions (75% ≥ hydrogen bond occupations > 50%) (iii) weak hydrogen bond interactions (50% ≥ hydrogen bond occupations > 25%) and (iv) very weak hydrogen bond interactions (25% ≥ hydrogen bond occupations > 10%) [29, 30, 48, 50].

## Results and discussion

### Identification of highly flexible regions

**Fig 2:**
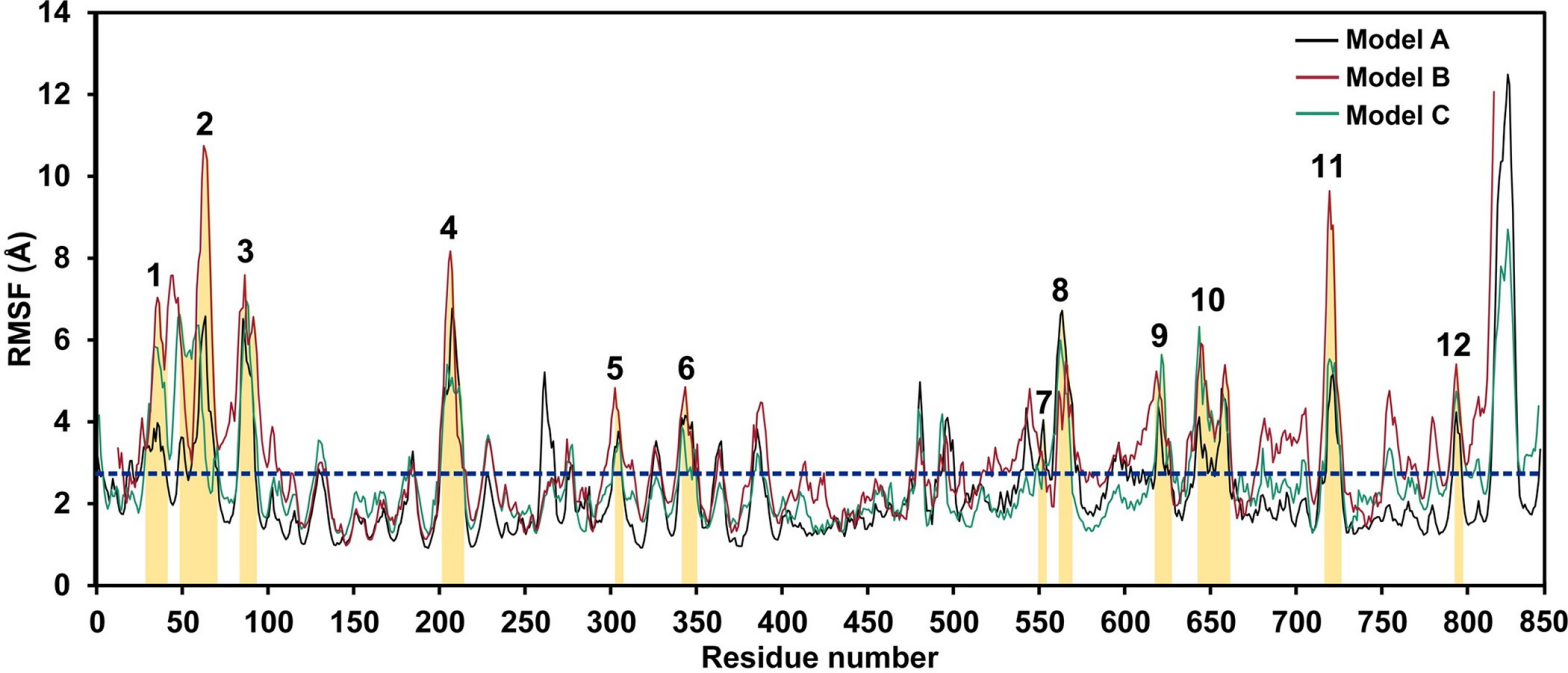
The RMSF plots of model A, B and C demonstrate highly flexible regions. The areas of twelve highly flexible regions were highlighted in orange. The cutoff value for identification of highly flexible regions is showed in blue dotted line.

MD was performed on the structures of wild-type rhamnosidase models at the optimum temperature of 313 K to allow structural relaxation and movements. The RMSD values of all atoms and backbone atoms were calculated to analyze the stabilities of all systems. As illustrated in S2 Fig in S2 File, all system were found to be quite stable in the range of 60 to 80 ns during the simulation period. Therefore, the structures from the 60–80 ns trajectory of each system were clustered to identify the centroid structure, which is a structure that is the most similar to the average structure. The centroid structure was selected to be a presentative structure of each model and subsequently simulated at high temperature (500 K) for 10 ns to increase structural movements to effectively identify highly flexible regions with high RMSF values. As shown in Fig 2, twelve highly flexible regions were identified in model A, B and C. The residues 815–843 were not classified as highly flexible residues because they are located at the C-terminus, which generally has high flexibility. Region 3 has the highest average RMSF value (5.5 Å), followed by regions 2 (5.1 Å), 8 (5.0 Å), 11 (4.9 Å) and 4 (4.9 Å), respectively (Table 1), suggesting that these regions may be essential for structural unfolding and the activity loss at high temperature. Therefore, increasing rigidity and favorable interactions of these regions can be a promising design strategy to improve the thermostability of this enzyme.

**Table 1:** Number of residues and average RMSF values of highly flexible regions of the wild-type rhamnosidase.

Region	Range of residues	Number of residues	Average RMSF (Å)
1	28–41	14	4.5 ± 0.5
2	48–70	23	5.1 ± 0.7
3	83–93	11	5.5 ± 0.5
4	201–214	14	4.9 ± 0.2
5	302–307	6	3.5 ± 0.3
6	341–350	10	3.6 ± 0.2
7	549–554	6	3.1 ± 0.2
8	561–569	9	5.0 ± 0.4
9	617–627	11	3.8 ± 0.3
10	642–661	20	4.2 ± 0.3
11	716–726	11	4.9 ± 0.9
12	792–797	6	4.1 ± 0.3

### Computational protein design

**Fig 3:**
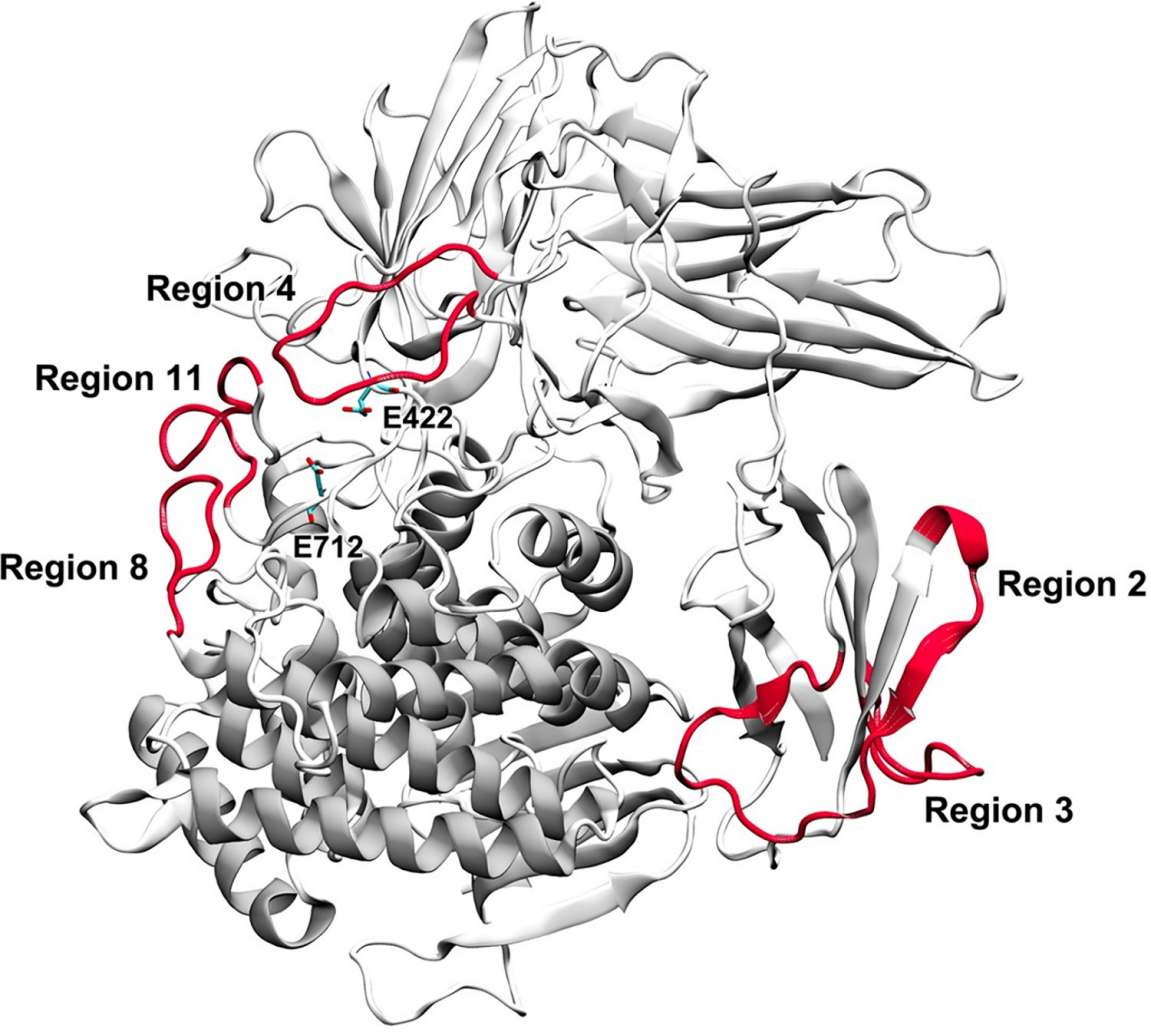
The centroid structure of Model A shows five highly flexible regions (region 2, 3, 4, 8 and 11) with the highest average RMSF value as shown in red secondary structure.

The centroid structure of model A (Fig 3) of the molecular dynamics trajectory was employed as a designed template because it has the highest coverage of the structure of α-L-Rhamnosidase from *Lactobacillus acidophilus*, as compared to Model B and C. Since a highly flexible region can disrupt favorable interactions of its neighboring residues, the structural stability of the enzyme can be improved by enhancing interactions of the highly flexible region with surrounding residues. Residues in the highly flexible regions that can potentially form favorable interactions with its neighboring residues, upon mutations, and are more than 15 Å away from the catalytic residues were selected as designed residues to avoid detrimental effects of mutations on enzymatic activity. The CoupledMoves protocol was used to design each highly flexible residue by allowing it to be any of standard amino acids that could potentially form favorable interactions with their neighboring residues or could increase interactions of neighboring residues. For each designed position, the designed mutants with the best values of ΔΔG and ΔΔG < 0 REU were selected for further analyses. Out of 57 designed positions from five highly flexible regions, Rosetta gave the total of 21 designed mutants with the best values of ΔΔG, where their ΔG values are also better than that of the wild type (ΔΔG < 0 REU). Eight, three, seven, two and one designed mutants from highly flexible regions 2, 3, 4, 8 and 11, respectively, were further simulated at high temperature (500K) to determine whether their highly flexible regions have lower flexibility than the wild type (Table 2).

**Table 2:** The ΔΔG values of the designed mutants.

Region	Residue number	Distance from the active site (Å)	Native amino acid	Have potential to interact with neighboring residues	Designed Mutant	ΔΔG (REU)
**2**	48	51.2	E	Yes	E	-
	49	52.8	S	Yes	E	-2.4
	50	53.0	E	No	-	-
	51	55.0	Q	No	-	-
	52	52.8	P	Yes	L	-3.8
	53	50.7	I	Yes	I	-
	54	49.5	Y	Yes	F	3.2
	55	52.9	F	Yes	V	-3.7
	56	48.4	E	Yes	H	-6.1
	57	51.5	P	Yes	G	-5.7
	58	53.8	D	Yes	D	-
	59	49.2	E	Yes	E	-
	60	49.9	L	Yes	L	-
	61	47.3	Y	Yes	L	3.8
	62	43.0	E	Yes	M	1.4
	63	40.9	N	Yes	W	1.0
	64	38.2	N	Yes	G	-5.9
	65	37.6	A	Yes	A	-
	66	41.3	F	Yes	W	2.7
	67	43.1	K	Yes	H	-4.5
	68	41.4	I	Yes	M	3.1
	69	45.2	N	Yes	W	-4.3
	70	45.0	M	Yes	M	-
**3**	83	49.9	G	Yes	G	-
	84	53.4	V	Yes	V	-
	85	57.2	R	Yes	R	-
	86	59.6	N	No	-	-
	87	59.9	D	No	-	-
	88	55.3	N	Yes	Q	-6.5
	89	57.8	E	Yes	E	-
	90	52.3	V	Yes	V	-
	91	51.0	T	Yes	I	-5.4
	92	49.9	S	Yes	S	-
	93	46.0	S	Yes	A	-4.1
**4**	201	21.9	G	Yes	G	-
	202	19.7	N	Yes	V	-2.1
	203	18.5	L	Yes	L	-
	204	15.2	G	Yes	G	-
	205	16.8	F	Yes	F	-
	206	14.2	D	Yes	-	-
	207	18.5	G	Yes	D	-1.6
	208	22.2	G	Yes	G	-
	209	22.0	Q	Yes	M	-1.5
	210	22.6	T	Yes	S	-2.6
	211	20.4	N	Yes	T	-3.5
	212	24.1	I	Yes	T	-2.2
	213	26.5	Y	Yes	K	-7.9
	214	26.4	G	Yes	G	-
**8**	561	16.7	N	Yes	A	1.0
	562	15.6	G	Yes	W	0.1
	563	14.4	A	Yes	-	-
	564	10.5	N	Yes	-	-
	565	13.9	P	No	-	-
	566	15.4	Q	Yes	Q	-
	567	19.1	G	No	A	1.3
	568	19.8	K	Yes	Q	-2.7
	569	23.7	T	Yes	G	-4.8
**11**	716	12.0	S	Yes	-	-
	717	16.3	V	Yes	V	-
	718	16.8	L	Yes	L	-
	719	19.8	P	Yes	H	-0.8
	720	18.6	D	Yes	D	-
	721	14.5	G	Yes	-	-
	722	14.2	K	Yes	-	-
	723	8.9	M	Yes	-	-
	724	11.2	N	Yes	-	-
	725	11.9	P	No	-	-
	726	8.6	E	Yes	-	-

### Validation by MD

MD simulations were performed on the structures of 21 designed mutants at 500 K in triplicates, and the RMSF values of the highly flexible regions of the selected designed mutants were analyzed. As shown in Table 3 and S3 Fig in S2 File, we found that the flexibilities of the designed regions of 14 designed mutants are lower than or similar to those of the wild type. In terms of the flexibilities of designed mutants in region 2, the flexibility of the designed region of the N64G mutant is lower than that of the wild type with the average ΔRMSF values of -9.1 ± 4.7%, while that of S49E (the average ΔRMSF values of -2.5 ± 5.8%) is about the same as that of the wild type. In terms of region 3, the flexibility of the designed region of N88Q is significantly lower than that of the wild type with the average ΔRMSF values of -18.9 ± 7.1%, while those of T91I and S93A mutants are similar to that of the wild type with the average ΔRMSF values of -0.8 ± 4.7% and -0.6 ± 4.1%, respectively. For region 4, the flexibilities of the designed region of all designed mutants (N202V, G207D, Q209M, T210S, N211T, I212T and Y213K mutants) are lower than that of the wild type with the average ΔRMSF values of -25.4 ± 1.1, -30.6 ± 1.2, -19.4 ± 3.0, -3.5 ± 2.3, -13.8 ± 1.6, -3.0 ± 0.9 and -12.7 ± 0.9%, respectively. In terms of region 8, the flexibilities of the designed region of K568Q and T569G mutants are also significantly lower than that of the wild type with the average ΔRMSF values of -15.6 ± 4.0 and -17.5 ± 4.3%, respectively. For region 11, the flexibility of the designed region of P719H mutant is higher than that of the wild type. The eight designed mutants with ΔRMSF values of the highly flexible regions lower than -10.0% (N88Q, N202V, G207D, Q209M, N211T, Y213K, K568Q and T569G mutants) were further simulated at the optimum temperature of the wild type (313 K) in triplicates to determine whether their designed regions, which are highly flexible regions, have lower flexibilities than those of the wild type at its optimum temperature.

**Table 3:** The average ΔRMSF of designed mutants after simulations at 500 and 313 K.

Region	Designed mutant	Average ΔRMSF (%)	
		500 K	313 K
2	S49E	-2.5 ± 5.8	-
	P52L	6.7 ± 5.2	-
	F55V	0.2 ± 4.7	-
	E56H	3.3 ± 5.8	-
	P57G	14.0 ± 5.9	-
	N64G	-9.1 ± 4.7	-
	K67H	14.7 ± 4.6	-
	N69W	14.9 ± 6.8	-
3	N88Q	-18.9 ± 7.1	-13.6 ± 10.2
	T91I	-0.8 ± 4.7	-
	S93A	-0.6 ± 4.1	-
4	N202V	-25.4 ± 1.1	-61.4 ± 7.2
	G207D	-30.6 ± 1.2	-58.0 ± 7.1
	Q209M	-19.4 ± 3.0	-54.5 ± 5.3
	T210S	-3.5 ± 2.3	-
	N211T	-13.8 ± 1.6	-56.0 ± 5.2
	I212T	-3.0 ± 0.9	-
	Y213K	-12.7 ± 0.9	-57.0 ± 5.8
8	K568Q	-15.6 ± 4.0	44.5 ± 16.4
	T569G	-17.5 ± 4.3	12.8 ± 12.5
11	P719H	15.7 ± 8.9	-

**Fig 4:**
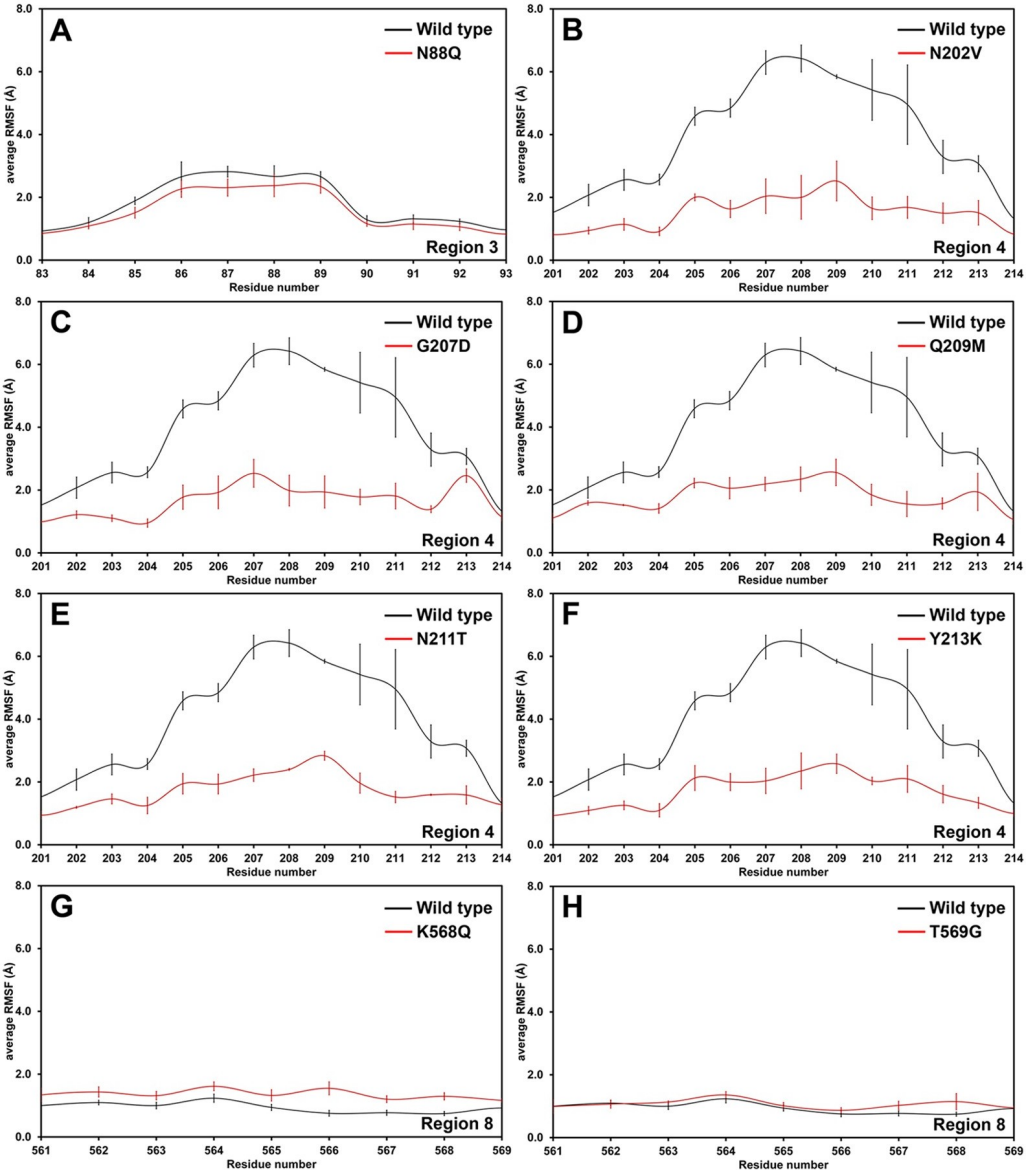
RMSF plots with their S.E.M. values of the designed mutants (A-H) as compared to those of the wild type after simulations at 313 K demonstrates that the flexibilities of the designed regions of N88Q, N202V, G207D, Q209M, N211T and Y213K mutants are lower than those of the wild type. Black lines are plots of the wild type, and red lines are plots of the designed mutants.

**Fig 5:**
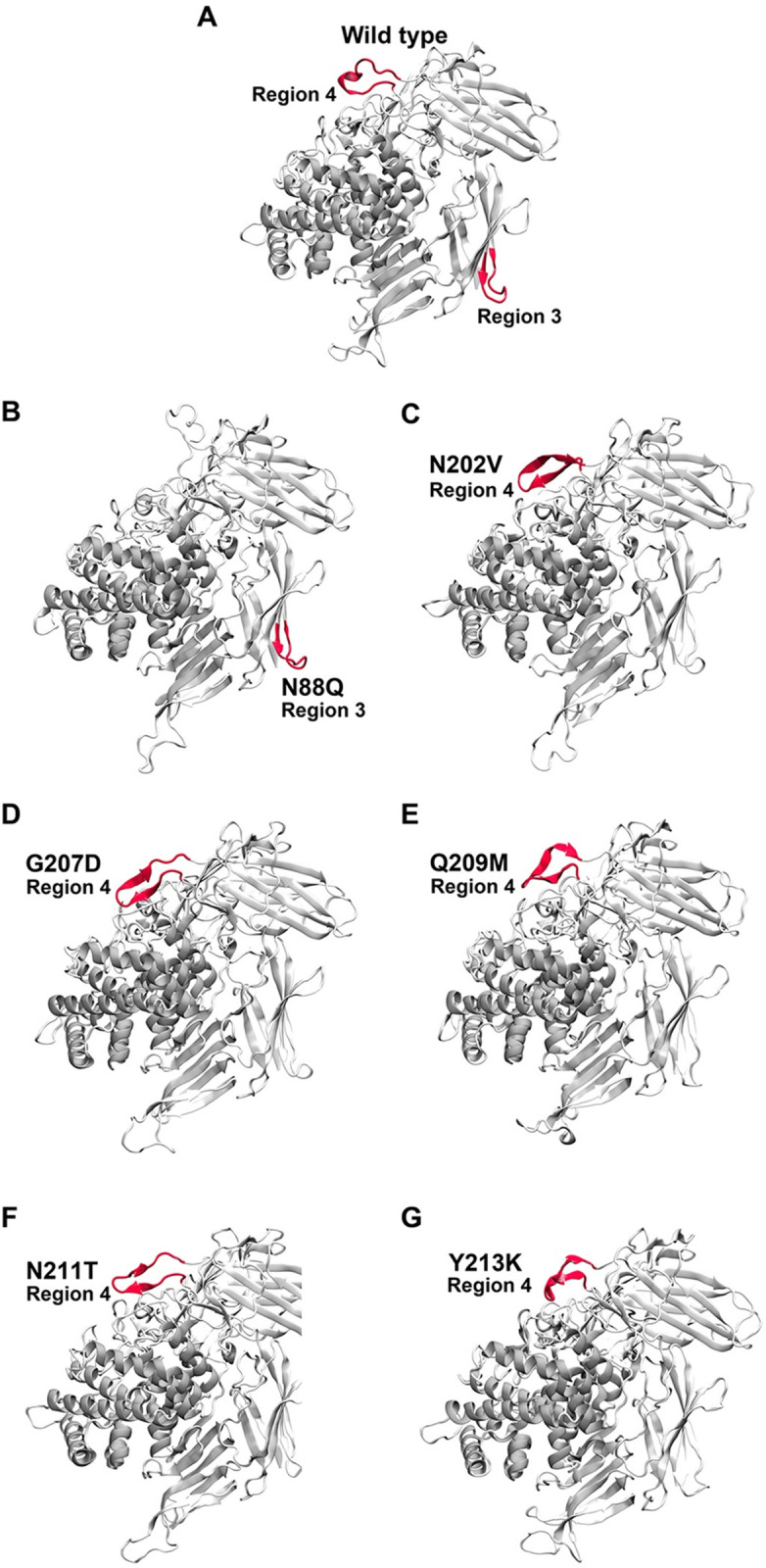
Overall structures of the wild type (A) and designed mutants (B-G) after simulations at 343K show the increased β-sheet structures in region 4 of N202V, G207D, Q209M, N211T and Y213K mutants.

The RMSD plots in S4 Fig in S2 File show that all system were found to be quite stable in the range of 60 to 80 ns during the simulation period at 313 K. Therefore, the 60–80 ns trajectory of each system was used for further analyses. The RMSF analysis shows that only the flexibilities of the designed regions of N88Q, N202V, G207D, Q209M, N211T and Y213K are lower than those of the wild type with the average ΔRMSF values of -13.6 ± 10.2, -61.4 ± 7.2, -58.0 ± 7.1, -54.5 ± 5.3, -56.0 ± 5.2 and -57.0 ± 5.8%, respectively (Table 3, Fig 4 and S5 Fig in S2 File). Additionally, Define Secondary Structure of Protein (DSSP) analysis was performed to elucidate the structural changes of the highly flexible regions of these designed mutants. As shown in Table 4 and Fig 5, the percentages of secondary structures of N88Q mutant in region 3 (β-sheet 32.8 ± 3.2%, helix 0.0 ± 0.0%, and random coil 67.1 ± 6.8%) are similar to those of the wild type (β-sheet 32.2 ± 4.3%, helix 0.0 ± 0.0%, and random coil 67.8 ± 9.2%), indicating that its overall structure is not significantly changed upon mutation. In terms of mutants with designed region 4, the percentages of β-sheet structures of all designed mutants are significantly increased from 0.2 ± 0.2% in the wild type to 20.2 ± 12.7, 19.8 ± 8.4, 21.8 ± 6.2, 12.0 ± 4.7 and 5.2 ± 3.6% in N202V, G207D, Q209M, N211T and Y213K mutants, respectively. However, the percentages of helix structures are decreased from 5.4 ± 4.5% in the wild type to 0.0 ± 0.0, 0.0 ± 0.0, 0.3 ± 0.3 and 4.1 ± 4.1% in N202V, G207D, Q209M and Y213K mutants, respectively, while that of N211T mutant (5.8 ± 5.7%) is about the same as that of the wild type. Overall, these results indicate that these five designed mutants were predicted to not only have lower flexibilities in region 4 than the wild type, but their percentages of β-sheet structures were also predicted to be higher than the wild type, suggesting that these mutants are promising designed enzymes with better structural stability than the wild type.

**Table 4:** Percentages of secondary structures of designed mutants, as calculated by Define Secondary Structure of Protein (DSSP).

Region	System	Percentages of secondary structures (%)		
		β-sheet	Helix	Random coil
3	Wild type	32.2 ± 4.3	0.0 ± 0.0	67.8 ± 9.2
	N88Q	32.8 ± 3.2	0.0 ± 0.0	67.1 ± 6.8
4	Wild type	0.2 ± 0.2	5.4 ± 4.5	94.4 ± 9.6
	N202V	20.2 ± 12.7	0.0 ± 0.0	79.8 ± 8.6
	G207D	19.8 ± 8.4	0.0 ± 0.0	80.2 ± 7.6
	Q209M	21.8 ± 6.2	0.3 ± 0.3	77.9 ± 7.2
	N211T	12.0 ± 4.7	5.8 ± 5.7	82.2 ± 5.5
	Y213K	5.2 ± 3.6	4.1 ± 4.1	90.7 ± 6.8

### Hydrogen bond interactions

**Fig 6:**
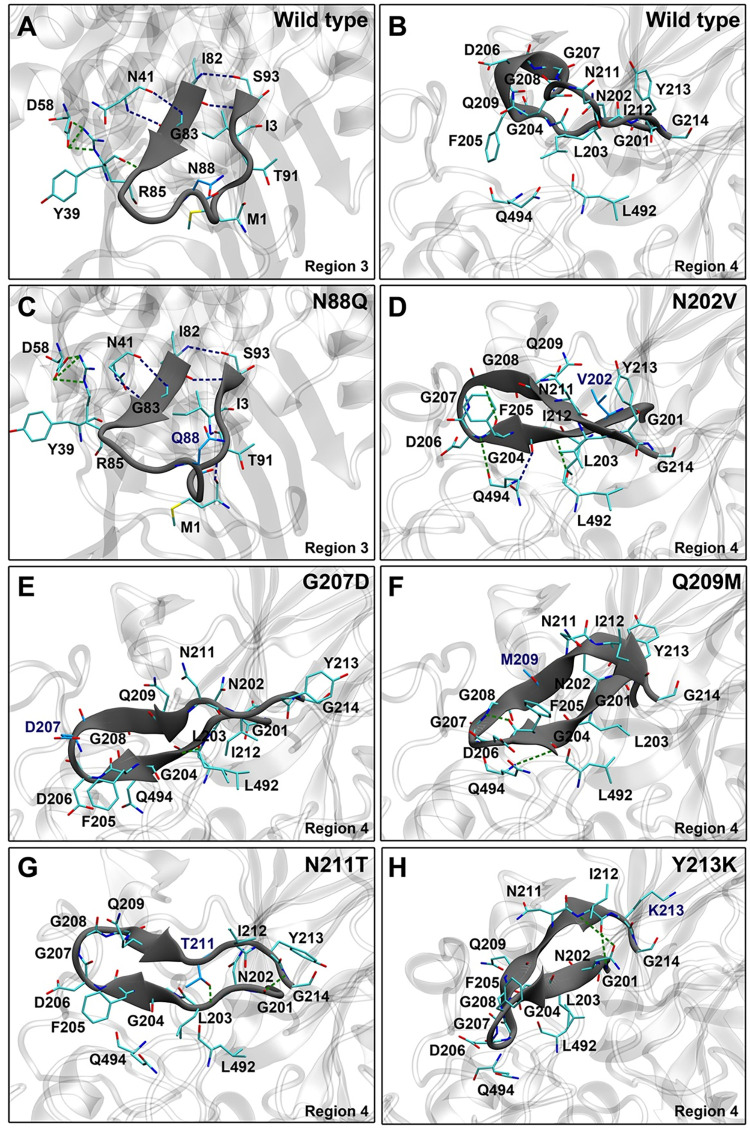
Overall hydrogen bond interactions of the designed regions of the wild type (A-B) are lower than those of the designed mutants (C-H). Strong and medium hydrogen bonds are shown in blue and green dashed lines, respectively.

Hydrogen bond interactions between the residues in the highly flexible regions and their neighboring residues of N88Q, N202V, G207D, Q209M, N211T and Y213K mutants were analyzed to obtain insight into how these designed mutants were predicted to have better structural stabilities than the wild type. As illustrated in Table 5, S1 Table in S2 File and Fig 6, the total number of predicted hydrogen bonds of N88Q mutant of region 3 is slightly lower than that of the wild type, but its number of strong hydrogen bonds (involving I82, G83, T91, and S93) is more than that of the wild type. Furthermore, the mutated residue Q88 was predicted to form two weak hydrogen bonds with the backbone of V84 and N86, while N88 of the wild type was predicted to form only one very weak hydrogen bond with E89.

**Table 5:** Number and strength of hydrogen bonds at 313 K of all residues in the highly flexible regions and their neighboring residues.

Region	System	Number of hydrogen bonds			
		Strong	Medium	Weak	Very weak
3	Wild type	4	4	5	10
	N88Q	6	3	5	3
4	Wild type	-	-	3	21
	N202V	1	3	9	17
	G207D	-	1	13	15
	Q209M	-	2	12	17
	N211T	-	2	12	17
	Y213K	-	2	7	25

In terms of hydrogen bonds of designed mutants of region 4, the total numbers of predicted hydrogen bonds of all designed mutants are substantially higher than those of the wild type. The N202V mutation was predicted to cause substantial increase in the total numbers of strong and medium hydrogen bonds involving G204, F205, D206 and G208, as compared to those of the wild type. Additionally. the mutated residue V202 was predicted to form three weak hydrogen bonds with I212 (backbone), Y213 (backbone) and N211, while N202 was predicted to form one weak hydrogen bond with the backbone of G204, and four very weak hydrogen bonds with D196, the backbones of F205, Y213 and G204.

The G207D mutation was predicted to form one medium hydrogen bond between the backbones of G204 and L492, while the wild type was not predicted to form any medium hydrogen bonds. The total number of weak hydrogen bonds of the G207D mutant was also predicted to be higher than that of the wild type. Furthermore, the mutated residue D207 was predicted to form three weak hydrogen bonds with Q209 and N211, while G207 of the wild type was predicted to form only one very weak hydrogen bond with the backbone of G204.

The Q209M mutant was predicted to have medium hydrogen bonds between the backbones of F205 and G208, and between the backbones of G204 and Q494, while the wild type was not predicted to form any medium hydrogen bonds. The Q209M mutation was also predicted to cause the increase in the total number of weak hydrogen bonds, as compared to that of the wild type. The mutated residue M209 was predicted to form one weak hydrogen bond with the backbone of F205, while Q209 of the wild type was predicted to form one weak hydrogen bond with the backbone of D206, and four very weak hydrogen bonds with the backbones of G204, F205 and T210. However, the Q209M mutation was predicted to cause substantial increase in the total number of hydrogen bonds between other residues in its region and their neighboring residues.

The N211T mutant was predicted to form two medium hydrogen bonds between the backbones of G201 and G214, and between the mutated residue T211 and the backbone of L203, while the wild type was not predicted to form any medium hydrogen bonds. Additionally, the mutated residue T211 was predicted to form two weak hydrogen bonds with N202, and four very weak hydrogen bonds with the backbones of L203, G204, F205 and I212, while N211 of the wild type was predicted to form three very weak hydrogen bonds with the backbones of L203 and G204.

The Y213K mutant was predicted to have two medium hydrogen bonds between the backbones of I212 and G201 and between the backbones of I212 and N202, while no medium hydrogen bond was predicted to form in the wild type. Moreover, the total numbers of weak and very weak hydrogen bonds of the Y213K mutant were also predicted to be higher than those of the wild type. Additionally, the mutated residue K213 was predicted to form three very weak hydrogen bonds with the backbone of D116, while Y213 of the wild type was predicted to form three very weak hydrogen bonds with the backbone of N202.

Overall, the mutated residue of each designed mutant could allow better packing of its residue and neighboring residues that increase the total numbers of predicted hydrogen bonds, resulting in improved favorable interactions and structural stability of the protein structure. N88Q, N202V, G207D, Q209M, N211T and Y213K are promising designed mutants that have high potential for stability improvement because the hydrogen bond interactions of the designed residues and their neighboring residues are better than those of the wild type.

## Conclusions

MD (AMBER) and computational protein design (Rosetta) techniques were employed to design mutants of α-L-rhamnosidase from *Lactobacillus acidophilus* to improve its structural stability by enhancing the rigidity and favorable interactions of residues in highly flexible regions with their neighboring residues. MD results show that five regions (2; 48–70, 3; 83–93, 4; 201–214, 8; 561–569 and 11; 716–726) have the highest flexibilities at high temperature (500 K) based on their RMSF values, and were selected for design. The residues in these designed regions were allowed to be any of standard amino acids to increase their rigidity and favorable interactions by Rosetta, and 21 designed mutants with the best values of ΔΔG, where their ΔG values are also better than that of the wild type, were obtained. To validate whether their designed regions have lower flexibility than the wild type, 21 designed mutants were simulated at high temperature (500 K), and eight promising designed mutants were further simulated at the optimum temperature of the wild type (313 K). MD results show that the flexibilities of the designed regions of N88Q, N202V, G207D, Q209M, N211T and Y213K are lower than those of the wild type. Additionally, these designed mutants were predicted to be more stable as they could maintain their native structures better than the wild type. The enhanced structural stabilities of these designed mutants are probably caused by the increased hydrogen bond interactions of the designed residues in highly flexible regions and their neighboring residues. This study designed N88Q, N202V, G207D, Q209M, N211T and Y213K mutants of *Lactobacillus acidophilus* α-L-rhamnosidase with better predicted structural stability than the wild type, and they are promising enzymes with high potential for stability improvement. It is worth noting that 80 ns simulations at 313 K were used in this study to preliminarily screen for promising mutants with high potential for stability improvement in reasonable amount of time for future experimental studies. Longer simulation time and further analysis techniques could also be used to improve the accuracy of our prediction and verify our findings.

## Supporting information

S1 File (PDF)

S2 File (RAR)
