# Supplementary material for: Computational design of Lactobacillus Acidophilus α-L-rhamnosidase to increase its structural stability
Source: PLoS One. 2022 May 25;17(5):e0268953. doi: 10.1371/journal.pone.0268953 (PMC9132286; doi:10.1371/journal.pone.0268953)
Supplement: S1 File — (PDF) [file pone.0268953.s001.pdf]

Computational design of *Lactobacillus Acidophilus* α-L-rhamnosidase to increase its structural stability

Thassanai Sitthiyotha[1], Methus Klaewkla[1], Kuakarun Krusong[1], Rath Pichyangkura[2] and Surasak Chunsrivirot[1*]

[1]Structural and Computational Biology Research Unit, Department of Biochemistry, Faculty of Science, Chulalongkorn University, Pathumwan, Bangkok 10330, Thailand

[2]Department of Biochemistry, Faculty of Science, Chulalongkorn University, Pathumwan, Bangkok 10330, Thailand.

*Corresponding Author

Email: surasak.ch@chula.ac.th

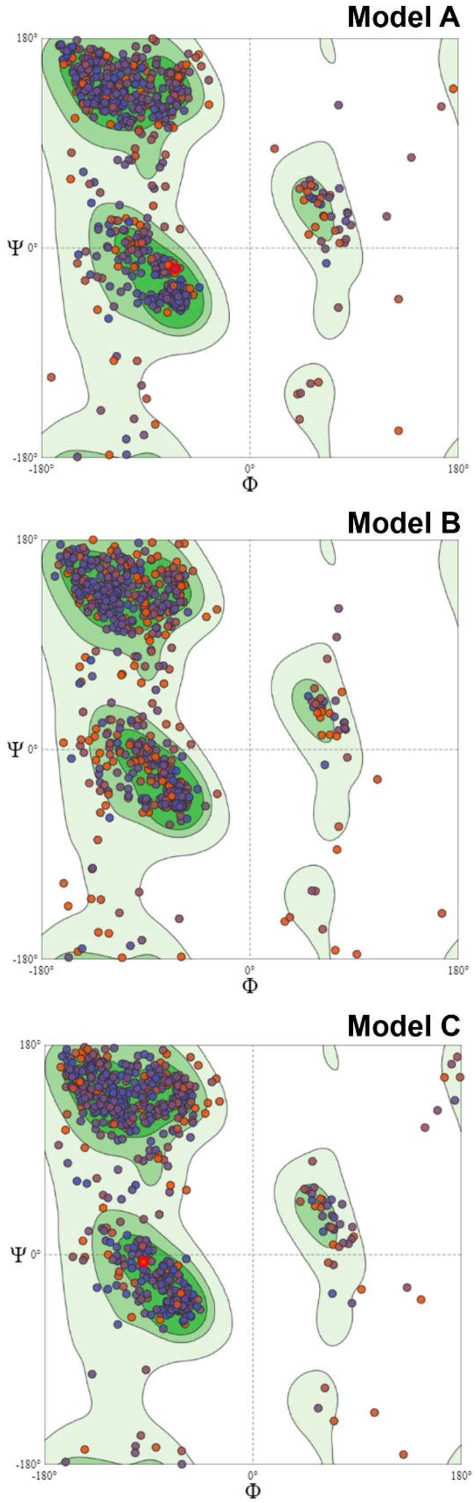

S1 Fig. Ramachandran plots of models A, B and C.

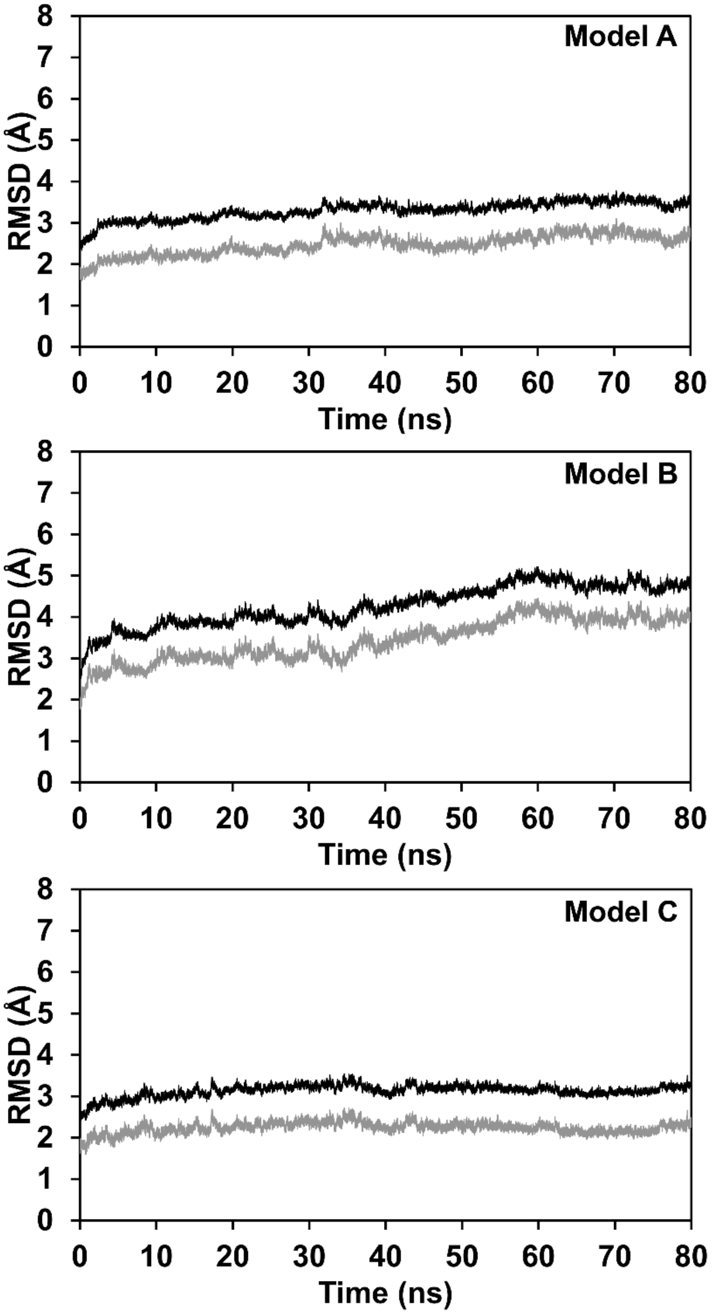

S2 Fig. RMSD plots of models A, B and C. The RMSD values of all atoms and backbone atoms are shown in black and gray, respectively.

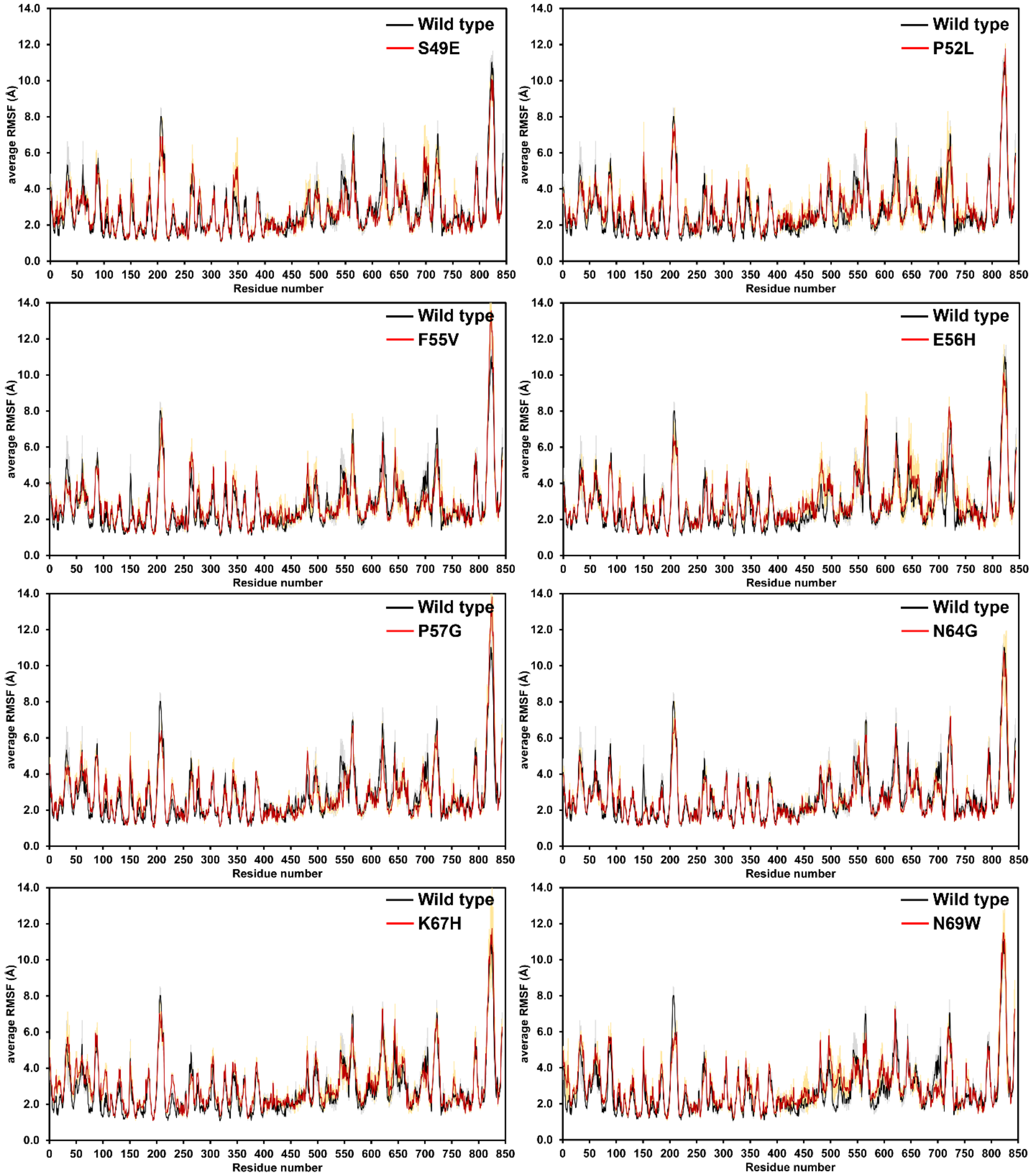
Wild type
S49E
Wild type
P52L
Wild type
F55V
Wild type
E56H
Wild type
P57G
Wild type
N64G
Wild type
K67H
Wild type
N69W
average RMSF (Å)
Residue number
0.0
2.0
4.0
6.0
8.0
10.0
12.0
14.0
0
50
100
150
200
250
300
350
400
450
500
550
600
650
700
750
800
850

19

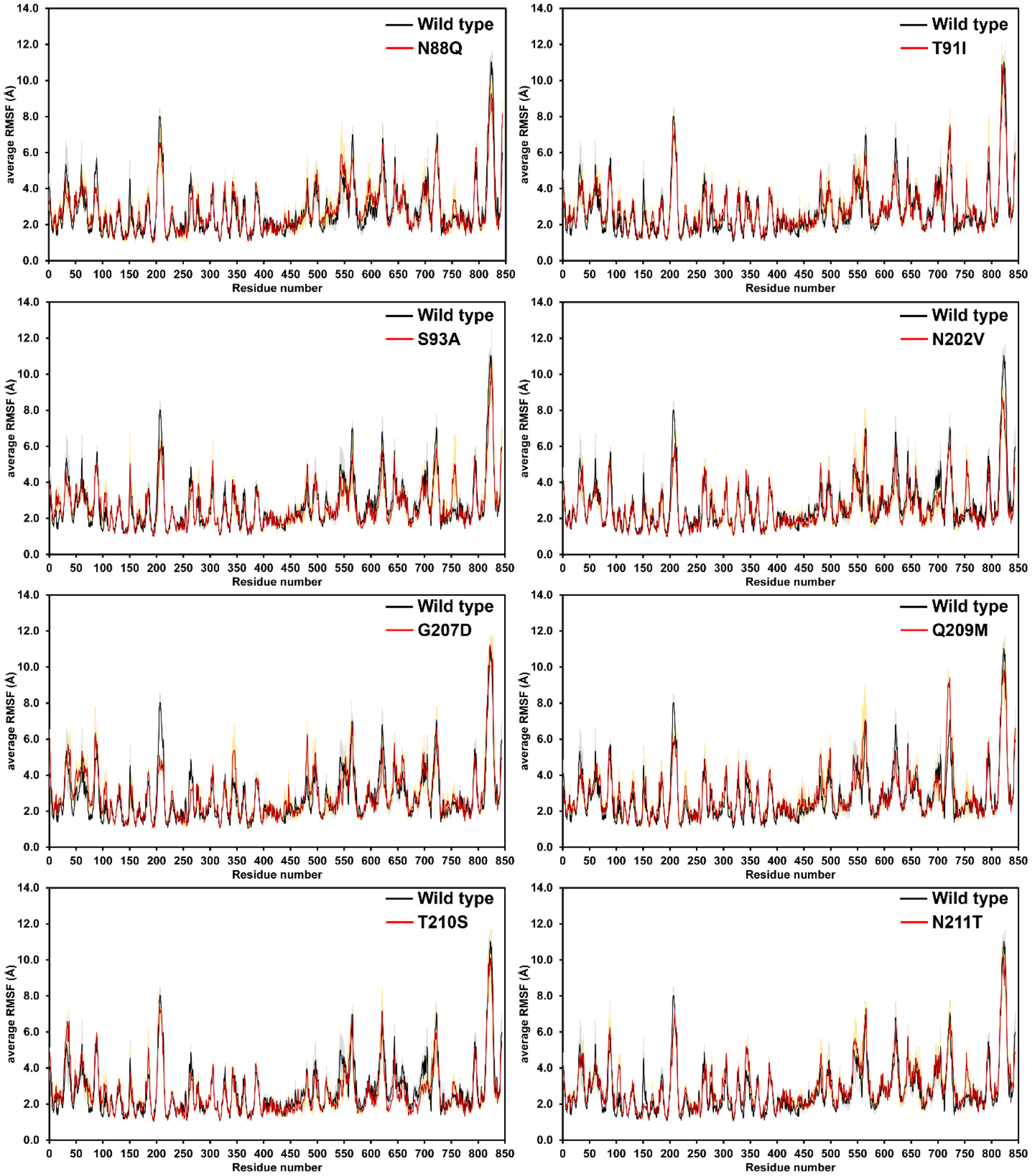
Wild type
N88Q
T91I
S93A
N202V
G207D
Q209M
T210S
N211T
average RMSF (Å)
Residue number
14.0
12.0
10.0
8.0
6.0
4.0
2.0
0.0
0 50 100 150 200 250 300 350 400 450 500 550 600 650 700 750 800 850

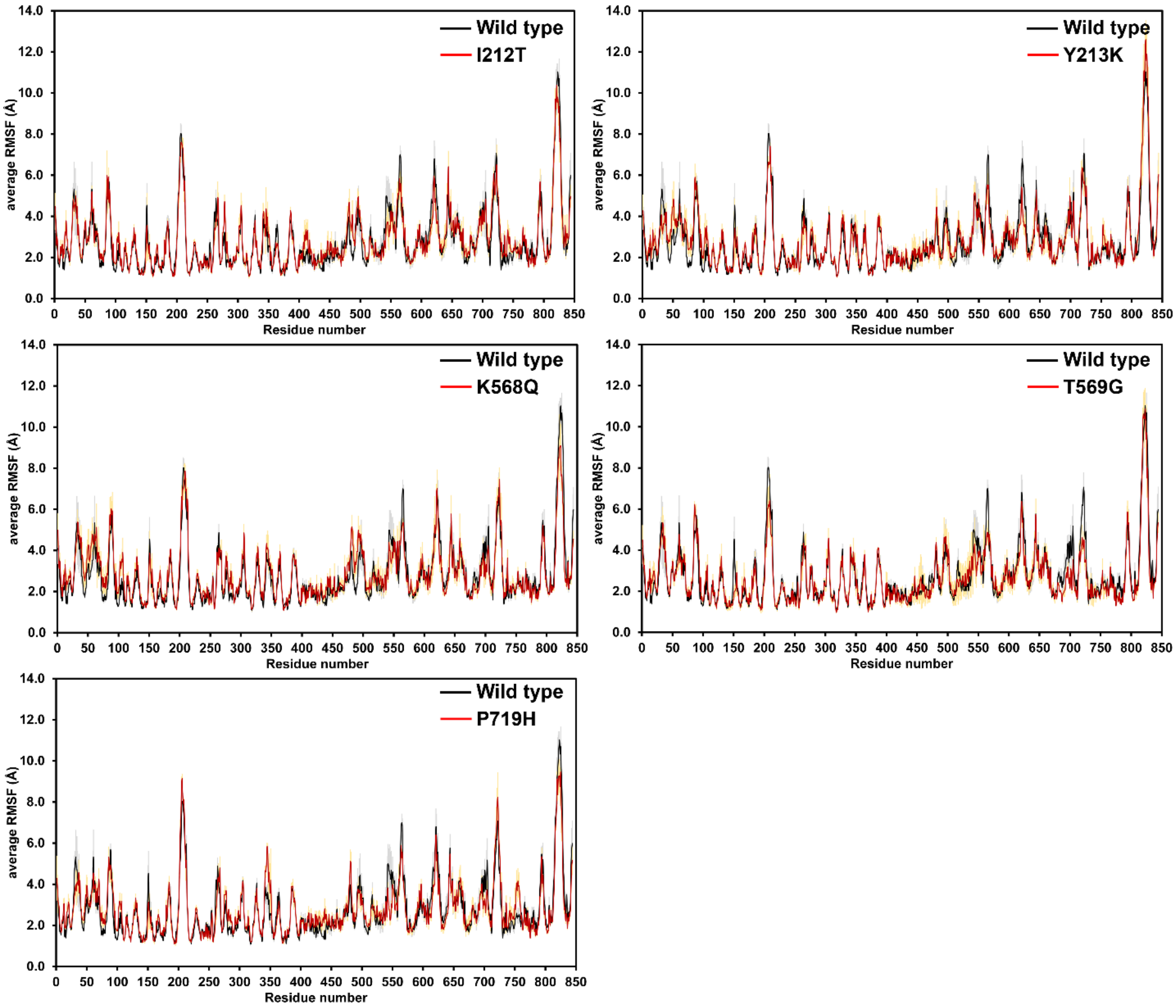

S3 Fig. RMSF plots with their S.E.M. values (shaded areas) of the designed mutants as compared to those of the wild type at 500 K. Black lines are plot of the wild type, and red line are plots of the designed mutants.

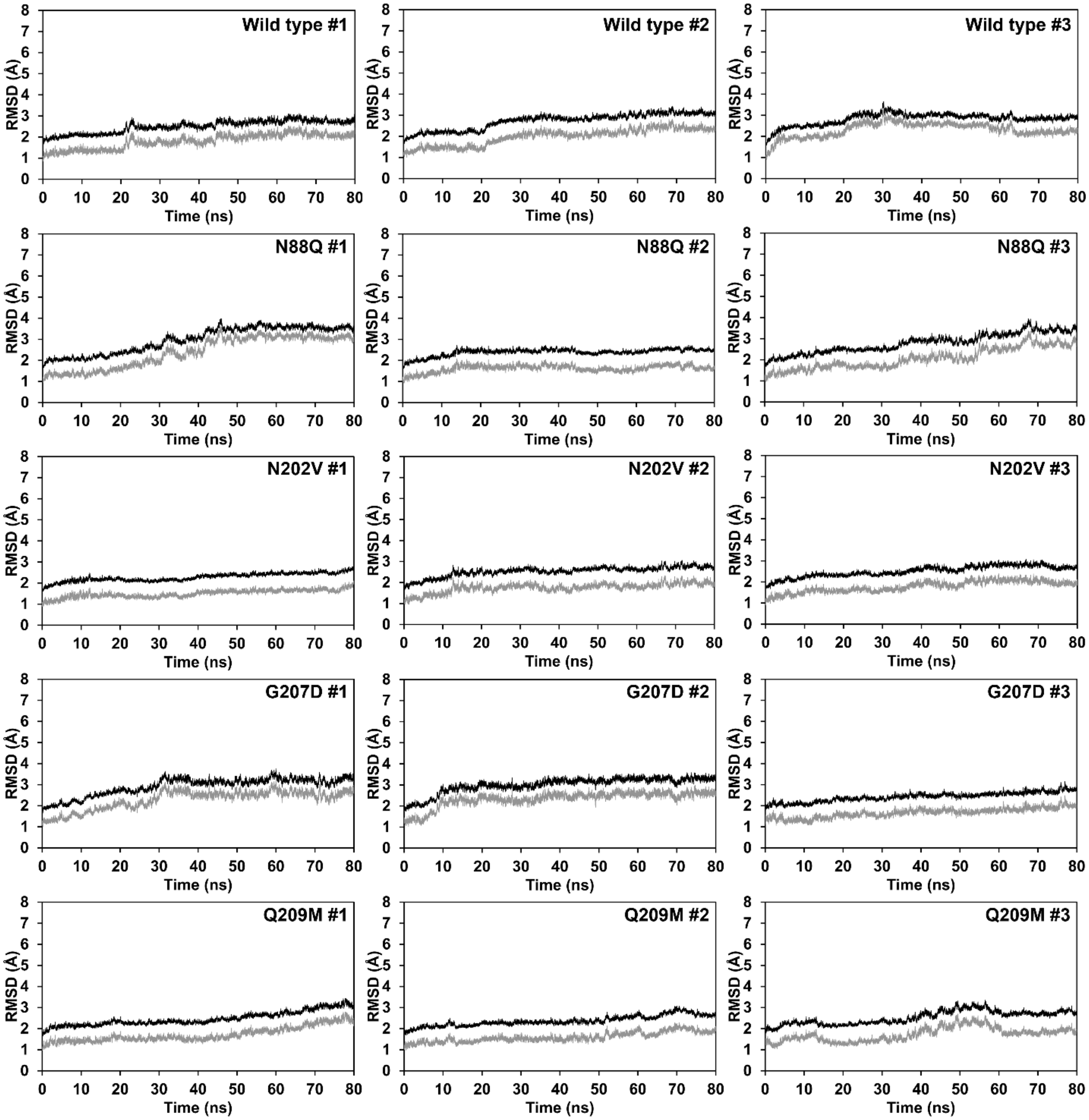
Wild type #1
Wild type #2
Wild type #3
N88Q #1
N88Q #2
N88Q #3
N202V #1
N202V #2
N202V #3
G207D #1
G207D #2
G207D #3
Q209M #1
Q209M #2
Q209M #3
RMSD (Å)
Time (ns)

32

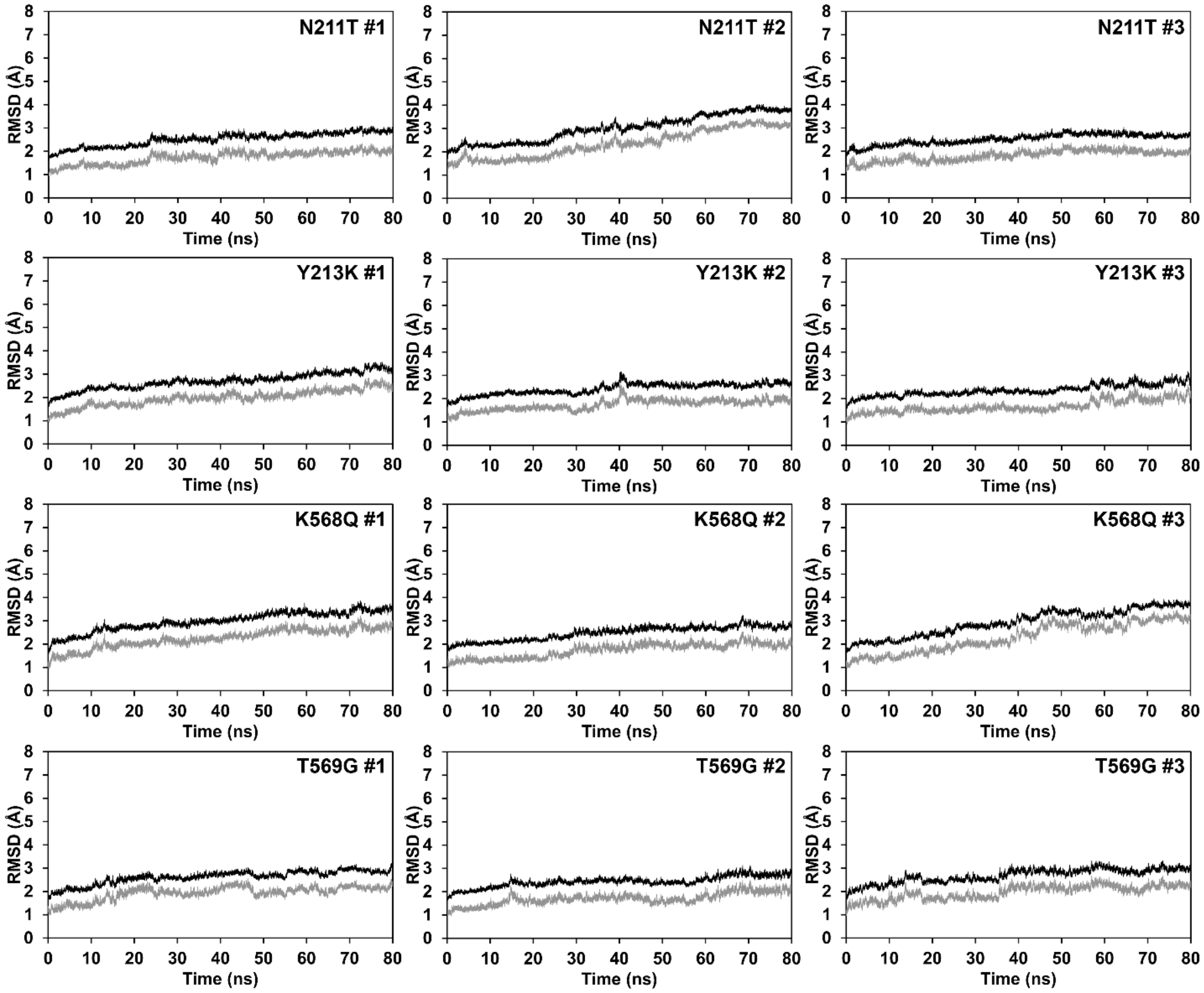

S4 Fig. RMSD plots of the wild type and designed mutants at 313 K. The RMSD values of all atoms and backbone atoms are shown in black and gray, respectively.

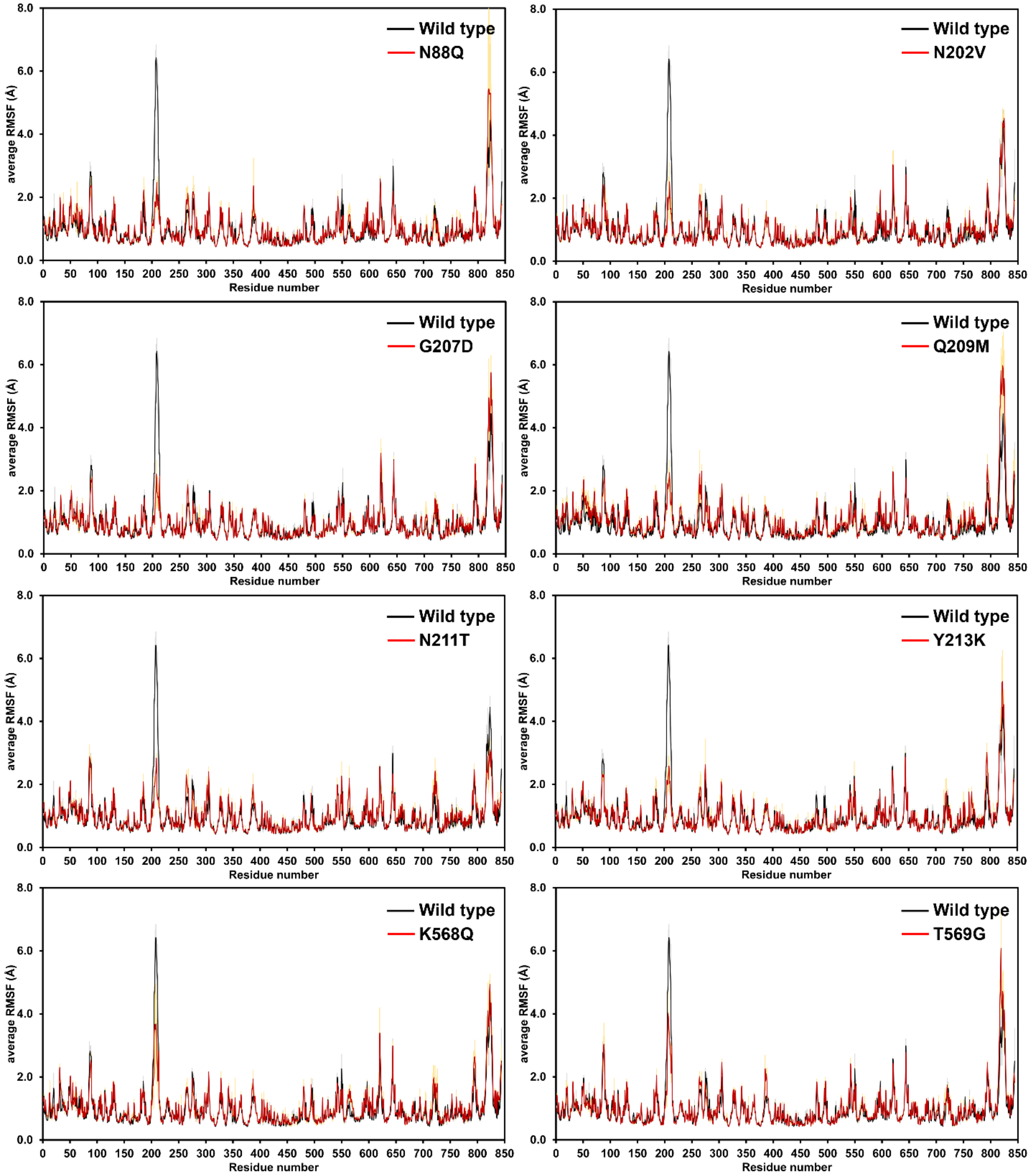

S5 Fig. RMSF plots with their S.E.M. values (shaded areas) of the designed mutants as compared to those of the wild type at 313 K. Black lines are plot of the wild type, and red line are plots of the designed mutants.

S1 Table. Hydrogen bond occupations of the wild type and designed mutants

System	Region	Acceptor	DonorH	Donor	Hydrogen bond occupancy (%)
Wild type	3	G83@O	N41@H	N41@N	98.3
		N41@O	G83@H	G83@N	95.0
		S93@O	I82@H	I82@N	88.2
		I82@O	S93@H	S93@N	76.5
		Y39@O	R85@H	R85@N	74.0
		D58@OD2	R85@HH11	R85@NH1	62.0
		D58@OD1	R85@HE	R85@NE	60.5
		D58@OD1	R85@HH11	R85@NH1	54.4
		T91@O	I3@H	I3@N	50.0
		D58@OD2	R85@HE	R85@NE	44.2
		M1@O	T91@H	T91@N	43.6
		I82@O	S92@HG	S92@OG	30.1
		N86@OD1	R85@HH22	R85@NH2	29.5
		R85@O	Y39@H	Y39@N	22.6
		S93@OG	N94@H	N94@N	19.6
		D87@O	E89@H	E89@N	18.1
		E89@OE2	E89@H	E89@N	17.8
		E89@OE2	D87@H	D87@N	16.9
		D87@O	V90@H	V90@N	15.9
		E89@OE2	N86@H	N86@N	15.7
		E89@OE1	N86@H	N86@N	13.0
		E89@OE2	N88@H	N88@N	12.6
		D87@OD2	E89@H	E89@N	10.5
	4	F205@O	G208@H	G208@N	40.0
		N202@OD1	G204@H	G204@N	29.6
		D206@O	Q209@H	Q209@N	25.7
		D196@OD2	N202@HD22	N202@ND2	22.4
		G204@O	G207@H	G207@N	21.9
		N202@O	F205@H	F205@N	21.6
		D206@O	T210@H	T210@N	20.3
		L203@O	Q494@H	Q494@N	18.1
		N211@OD1	L203@H	L203@N	17.2
		G204@O	N211@HD21	N211@ND2	16.1
		D206@OD2	G208@H	G208@N	15.8
		Y213@O	N202@H	N202@N	14.9
		T210@O	I212@H	I212@N	14.5
		N202@O	G204@H	G204@N	14.4
		N211@OD1	G204@H	G204@N	13.9
		F205@O	I212@H	I212@N	12.7
		G201@O	G214@H	G214@N	12.6
		D206@O	Q494@H	Q494@N	12.5
		D206@OD1	G208@H	G208@N	12.3
		Q209@OE1	T210@H	T210@N	12.2
		Y255@OH	D206@H	D206@N	11.3
		F205@O	Q209@H	Q209@N	11.2
		G204@O	Q209@H	Q209@N	11.0
		T210@O	Q209@HE21	Q209@NE2	10.4

System	Region	Acceptor	DonorH	Donor	Hydrogen bond occupancy (%)
N88Q	3	G83@O	N41@H	N41@N	99.4
		S93@O	I82@H	I82@N	97.8
		N41@O	G83@H	G83@N	96.1
		T91@O	I3@H	I3@N	90.1
		I82@O	S93@H	S93@N	89.0
		M1@O	T91@H	T91@N	77.0
		D58@OD2	R85@HH11	R85@NH1	69.0
		D58@OD1	R85@HH11	R85@NH1	68.0
		D58@OD1	R85@HE	R85@NE	62.2
		D58@OD2	R85@HE	R85@NE	50.0
		Y39@O	R85@H	R85@N	42.9
		D87@O	E89@H	E89@N	41.4
		N86@O	Q88@HE21	Q88@NE2	29.9
		V84@O	Q88@HE22	Q88@NE2	27.0
		D87@O	V90@H	V90@N	22.5
		S93@OG	N94@H	N94@N	19.8
		I82@O	S92@HG	S92@OG	19.6
N202V	4	G204@O	Q494@H	Q494@N	97.8
		L492@O	G204@H	G204@N	68.7
		F205@O	G208@H	G208@N	65.4
		Q494@O	D206@H	D206@N	51.0
		I212@O	V202@H	V202@N	33.5
		L203@O	T210@H	T210@N	32.5
		G208@O	F205@H	F205@N	32.5
		D196@OD1	Y213@HH	Y213@OH	32.3
		Y213@O	V202@H	V202@N	31.5
		G201@O	G214@H	G214@N	30.7
		V202@O	N211@HD22	N211@ND2	29.5
		Y199@OH	G214@H	G214@N	26.9
		L492@O	N211@HD21	N211@ND2	26.1
		L203@O	T210@HG1	T210@OG1	24.9
		Q209@O	Y255@HH	Y255@OH	24.9
		I212@O	L203@H	L203@N	24.0
		T210@OG1	I212@H	I212@N	21.4
		N211@OD1	N211@H	N211@N	19.2
		D206@OD2	S496@H	S496@N	18.9
		K200@O	I212@H	I212@N	15.8
		D206@OD2	S496@HG	S496@OG	15.8
		D206@OD1	K495@HZ1	K495@NZ	15.2
		D206@OD1	K495@HZ3	K495@NZ	13.9
		D206@OD1	K495@HZ2	K495@NZ	13.8
		E726@OE1	Q209@HE21	Q209@NE2	13.2
		F205@O	G207@H	G207@N	12.8
		D206@OD2	K495@HZ1	K495@NZ	12.1
		D206@OD1	S496@H	S496@N	11.9
		D206@OD1	S496@HG	S496@OG	10.7
		D206@OD2	K495@HZ2	K495@NZ	10.2
G207D	4	L492@O	G204@H	G204@N	64.1
		G204@O	Q494@H	Q494@N	42.9

System	Region	Acceptor	DonorH	Donor	Hydrogen bond occupancy (%)
		N202@OD1	I212@H	I212@N	40.4
		L203@O	N202@HD22	N202@ND2	35.9
		Q209@OE1	Q209@H	Q209@N	33.9
		L203@O	T210@H	T210@N	32.7
		Q209@OE1	D207@H	D207@N	32.6
		N211@OD1	N202@HD21	N202@ND2	32.4
		S491@O	G204@H	G204@N	32.3
		I212@O	L203@H	L203@N	31.7
		G208@O	F205@H	F205@N	31.7
		G201@O	G214@H	G214@N	31.7
		I212@O	G201@H	G201@N	31.3
		Q209@OE1	D206@H	D206@N	25.6
		F205@O	G208@H	G208@N	24.7
		L203@O	T210@HG1	T210@OG1	22.4
		T210@O	Q494@HE22	Q494@NE2	20.0
		G204@O	Q494@HE21	Q494@NE2	19.7
		Q494@O	D206@H	D206@N	18.3
		D207@O	Q209@H	Q209@N	18.2
		T210@OG1	L203@H	L203@N	16.8
		I212@O	N202@HD22	N202@ND2	15.6
		T210@O	I212@H	I212@N	15.1
		T210@O	N202@HD21	N202@ND2	14.9
		N211@OD1	N202@HD22	N202@ND2	12.7
		G214@O	K217@HZ3	K217@NZ	11.7
		D206@OD1	T497@HG1	T497@OG1	11.5
		G214@O	K217@HZ2	K217@NZ	11.4
		K200@O	N202@H	N202@N	10.2
Q209M	4	F205@O	G208@H	G208@N	68.6
		G204@O	Q494@H	Q494@N	59.6
		Q494@O	D206@H	D206@N	46.1
		L492@O	N211@HD21	N211@ND2	33.5
		I212@O	G201@H	G201@N	32.9
		D215@O	Y213@HH	Y213@OH	32.5
		L203@O	T210@H	T210@N	32.0
		I212@O	N202@H	N202@N	31.7
		M209@O	F205@H	F205@N	31.5
		L492@O	G204@H	G204@N	30.4
		T210@OG1	L203@H	L203@N	28.4
		G201@O	L203@H	L203@N	28.2
		N211@OD1	G204@H	G204@N	27.2
		G208@O	F205@H	F205@N	26.5
		Q494@OE1	G207@H	G207@N	24.4
		G201@O	G214@H	G214@N	23.8
		T210@O	N202@HD22	N202@ND2	21.9
		I212@O	T210@HG1	T210@OG1	21.1
		G204@O	N211@HD21	N211@ND2	20.9
		N202@O	N211@HD22	N211@ND2	20.2
		I212@O	N202@HD22	N202@ND2	18.7
		G208@O	Q494@H	Q494@N	18.0

System	Region	Acceptor	DonorH	Donor	Hydrogen bond occupancy (%)
		N211@OD1	N202@HD22	N202@ND2	17.9
		N202@O	I212@H	I212@N	17.3
		Y199@O	G214@H	G214@N	16.4
		G201@O	N211@HD22	N211@ND2	14.8
		D206@O	G208@H	G208@N	14.2
		Y199@OH	G214@H	G214@N	12.5
		T210@O	I212@H	I212@N	12.1
		D196@OD1	Y213@HH	Y213@OH	10.5
		D168@OD1	N202@HD22	N202@ND2	10.2
N211T	4	G201@O	G214@H	G214@N	74.2
		T211@OG1	L203@H	L203@N	59.5
		Q209@O	F205@H	F205@N	45.4
		K200@O	N202@H	N202@N	34.4
		I212@O	L203@H	L203@N	32.9
		G201@O	D215@H	D215@N	32.5
		N202@OD1	T211@H	T211@N	32.3
		D206@O	Q494@H	Q494@N	32.2
		G207@O	T210@H	T210@N	31.4
		L492@O	D206@H	D206@N	31.2
		N202@OD1	G204@H	G204@N	30.8
		G204@O	Q494@H	Q494@N	30.7
		T211@O	N202@HD21	N202@ND2	29.7
		F205@O	N202@HD22	N202@ND2	28.4
		L203@O	T211@H	T211@N	24.0
		Y199@O	G201@H	G201@N	23.6
		L492@O	G204@H	G204@N	23.3
		G207@O	T210@HG1	T210@OG1	23.1
		N202@O	G204@H	G204@N	21.9
		N202@O	Y255@HH	Y255@OH	20.6
		L203@O	T210@HG1	T210@OG1	18.9
		I212@O	T211@HG1	T211@OG1	18.2
		Q209@O	G207@H	G207@N	15.2
		F205@O	G208@H	G208@N	15.1
		D206@OD1	G207@H	G207@N	15.0
		D206@OD1	T210@HG1	T210@OG1	12.4
		F205@O	T211@HG1	T211@OG1	12.2
		T211@OG1	G204@H	G204@N	10.9
		F205@O	T210@HG1	T210@OG1	10.3
		E118@OE1	Y213@HH	Y213@OH	10.3
		D206@O	Q209@H	Q209@N	10.1
Y213K	4	I212@O	G201@H	G201@N	62.5
		N202@OD1	I212@H	I212@N	55.9
		G204@O	Q494@H	Q494@N	48.9
		F205@O	G208@H	G208@N	42.2
		D168@OD2	L203@H	L203@N	31.9
		D168@OD1	N202@HD22	N202@ND2	31.8
		G208@O	G204@H	G204@N	30.6
		Y255@OH	N211@H	N211@N	28.9
		L203@O	Q494@HE21	Q494@NE2	28.8

System	Region	Acceptor	DonorH	Donor	Hydrogen bond occupancy (%)
		D196@OD2	G214@H	G214@N	21.5
		Q209@OE1	F205@H	F205@N	20.1
		Q494@O	D206@H	D206@N	18.5
		D215@OD2	N202@HD21	N202@ND2	18.0
		Q209@OE1	N202@HD22	N202@ND2	17.1
		D215@OD1	N202@HD21	N202@ND2	16.0
		T210@OG1	I212@H	I212@N	15.7
		D206@OD1	K495@HZ2	K495@NZ	15.0
		D206@OD1	K495@HZ3	K495@NZ	14.6
		N211@O	Y255@HH	Y255@OH	14.2
		G204@O	G208@H	G208@N	14.2
		D116@O	K213@HZ2	K213@NZ	14.0
		N202@ND2	Y199@HH	Y199@OH	13.6
		D206@OD1	K495@HZ1	K495@NZ	13.4
		D116@O	K213@HZ3	K213@NZ	12.9
		D206@OD1	S496@H	S496@N	12.6
		Q209@OE1	Q209@H	Q209@N	12.5
		D206@OD2	S496@H	S496@N	12.3
		N202@ND2	G204@H	G204@N	12.3
		G204@O	G207@H	G207@N	11.9
		D116@O	K213@HZ1	K213@NZ	11.6
		N202@OD1	N211@HD21	N211@ND2	11.4
		Q209@OE1	G204@H	G204@N	10.7
		N211@OD1	K200@HZ3	K200@NZ	10.3
		N202@O	N211@HD21	N211@ND2	10.3
